# Embryonic Development of *Appasus japonicus* Vuillefroy, With Special Reference to Mouthparts Formation (Insecta: Heteroptera, Belostomatidae)

**DOI:** 10.1002/jmor.70052

**Published:** 2025-05-14

**Authors:** Tomoya Suzuki, Takashi Tanizawa, Nobuo Suzuki, Koji Tojo

**Affiliations:** ^1^ Department of Biology, Tomoya Faculty of Science Shinshu University Nagano Japan; ^2^ Faculty of Human Environmental Studies Hiroshima Shudo University Hiroshima Japan; ^3^ Division of Environmental System Science, Graduate School of Science and Technology Shinshu University Nagano Japan; ^4^ Department of Movement Sciences, Faculty of Sports and Health Science Japan Women's College of Physical Education Tokyo Japan; ^5^ Institute of Mountain Science Shinshu University Nagano Japan

**Keywords:** embryology, Heteroptera, labial palp, maxillary palp, maxillary plate, mouthparts development

## Abstract

The order Hemiptera *s. lat*. (=Homoptera + Heteroptera), comprising approximately 140 families and 70,000 species, is the largest order among hemimetabolous insects in terms of species diversity. A key trait shared among these insects is their specialized piercing‐sucking mouthparts, which have been considered an important factor in their diversification. However, knowledge of how these characteristic hemipteran mouthparts form during embryogenesis remains limited and biased toward model species. In this study, we observed the embryonic development of the heteropteran insect *Appasus japonicus* (Belostomatidae). We divided its embryonic development into 10 stages and provided a detailed description. Additionally, we examined its developmental processes and compared them with the embryogenesis of closely related groups. As a result, we confirmed that (1) the maxillary plate, one of the structures forming the heteropteran mouthparts, is homologous to the maxillary palp, and (2) most parts of the stylet‐like mandibles and maxillae are housed within the labial palp.

## Graphical abstract

In this study, we provide a detailed description of the embryonic development of *Appasus japonicus*, focusing on the formation of its specialized mouthparts. We identify homologies between the maxillary plate and maxillary palp and most parts of the stylet‐like mandibles and maxillae are housed within the labial palp. These findings contribute to a deeper understanding of mouthparts evolution in Hemiptera.

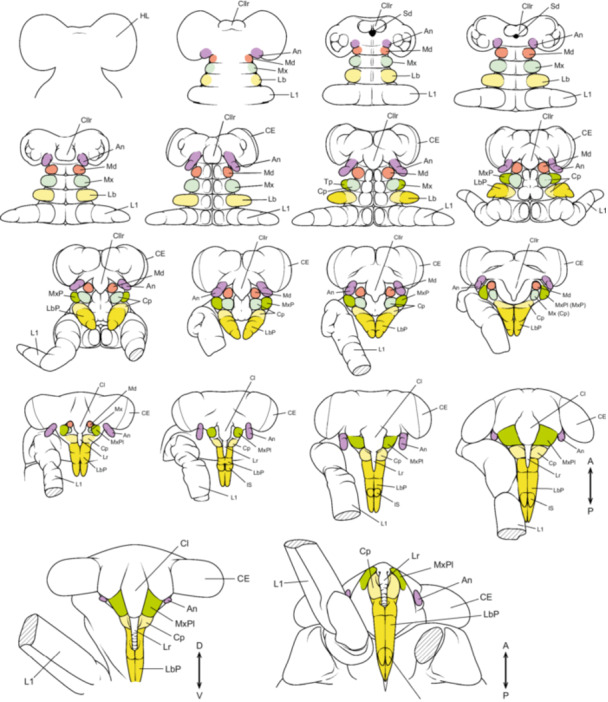

## 1 Introduction

**Figure 1:**
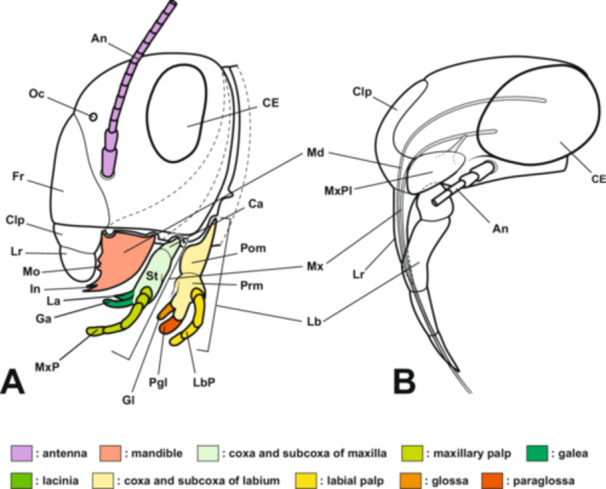
Scheme of the head in (A) a biting‐type insect (based on Snodgrass 1935) and (B) a hemipteran insect (based on the giant water bug *Appasus japonicus*). In Hemiptera, the labrum and labium form a tubular structure, while the mandibles and maxillae extend externally after passing through their interior. An, antenna; Ca, cardo; CE, compound eye; Clp, clypeus; Fr, frons; Ga, galea; Gl, glossa; In, incisor; La, lacinia; Lb, labium; LbP, labial palp; Lr, labrum; Md, mandible; Mo, molar; Mx, maxilla; MxP, maxillary palp; MxPl, maxillary plate; Oc, ocellus; Pgl, paraglossa; Pom, postmentum; Prm, prementum; St, stipes.

Hemiptera *s. lat*. (=Homoptera + Heteroptera) is the most species‐rich group among hemimetabolous insects (Tojo et al. 2017), having adapted to a wide range of environments, including terrestrial, freshwater, and marine habitats. Their morphology is also highly diverse, exemplified by the various head capsule forms of treehoppers, the specialized legs of water striders adapted for water surface, and the unique morphology of aquatic hemipterans that facilitates respiration in water. It is widely believed that their piercing‐sucking mouthparts (Figure 1B), a shared derived trait within the Hemiptera, have played a significant role in the diversification of this group (Yoshizawa and Saigusa 2003; Grimaldi and Engel 2005; Johnson et al. 2018; Weirauch et al. 2019). Many studies have provided insights into the functional modifications of these mouthparts (e.g., Cobben 1968; Muir and Kershaw 1911; Tull et al. 2020; Wang et al. 2020). However, only a few studies have addressed the embryonic development and evolutionary origins of the mouthparts in hemipteran insects (e.g., Dorn and Hoffmann 1983; Newcomer 1948; Rogers et al. 1997, 2002; Snodgrass 1935). Additionally, comparing the mouthparts of psocopteran and hemipteran insects is crucial, as Psocoptera represents the most basal group within the Paraneoptera and possesses biting mouthparts. From this perspective, several studies have conducted morphological observations on adult psocopteran insects (Yoshizawa and Saigusa 2003). Nevertheless, studies comparing the homologous relationship between the biting mouthparts of typical insects and the piercing‐sucking mouthparts of hemipteran insects through embryonic development are limited. Furthermore, the embryogenesis of hemipteran insects has primarily been studied in model species, such as the milkweed bug, *Oncopeltus fasciatus* (e.g., Angelini et al. 2005; Birkan et al. 2011; Hrycaj et al. 2008). In recent years, studies have identified genes involved in the mouthpart formation of the Neotropical brown stink bug, *Euschistus heros*. However, no studies have provided a detailed description of its morphogenetic processes (Cagliari et al. 2021).

Aquatic stink bugs have been suggested to represent relatively ancestral lineages within Heteroptera (Weirauch et al. 2019). Therefore, investigating the embryonic development of the aquatic stink bugs is expected to provide fundamental insights into the embryogenesis of Heteroptera. Molecular phylogenetic analyses have revealed that Homoptera constitutes a paraphyletic group in relation to Heteroptera, indicating that Homoptera represents a more ancestral lineage (Johnson et al. 2018). However, Heteroptera is the most species‐rich group among hemimetabolous insects (Li et al. 2012) and can therefore be regarded as the most evolutionarily successful lineage in terms of species diversity. Consequently, investigating the embryonic development of aquatic stink bugs, which represent an ancestral lineage within Heteroptera, is of considerable significance. In this study, we investigated the embryogenesis of the giant water bug, *Appasus japonicus*, an aquatic heteropteran insect, with a focus on external observations, particularly the formation of the mouthparts. We also discuss the evolution of the heteropteran piercing‐sucking mouthparts, which are considered one of the key factors driving the diversification of heteropteran insects.

## 2 Materials and Methods

Adults of *A. japonicus* Vuillefroy, 1864 (Insecta, Heteroptera, Belostomatidae) were collected in Matsumoto, Nagano Prefecture, Japan, between 2003 and 2008. Embryo fixation was carried out between 2003 and 2008, and subsequent experiments were conducted using the fixed samples. All collected specimens were brought to the laboratory and kept in plastic containers (365 × 215 × 250 mm) at room temperature, maintaining an almost 1:1 sex ratio. Female belostomatine insects lay their eggs on the male's back (primarily on the dorsal surface of the forewings), forming an egg mass, and the males care for the eggs until the eggs hatch. Mating and oviposition occurred in the plastic containers, and each male carrying an egg mass was transferred to a separate container (111 × 78 × 42 mm) and maintained at 20°C. Eggs were removed from the egg mass for observation of embryogenesis, and were punctured with a fine needle before being fixed in alcoholic Bouin's fluid (saturated alcoholic solution of picric acid: formalin: acetic acid = 15:5:1) or Karnovsky's fluid (2% paraformaldehyde + 2.5% glutaraldehyde) at room temperature for 24 h. The fixed eggs were then stored in 70% ethanol.

Some fixed embryos were stained with 0.01% Mayer's acid hemalum (Merck, Darmstadt) and observed using an SMZ1500 stereomicroscope (Nikon, Tokyo), following the method described by Tanizawa et al. (2007). We sketched the embryos observed with the stereomicroscope using the Adobe Illustrator software. The embryonic development of *A. japonicus* was divided into 10 stages, following the classification system of Tanaka (2001).

For scanning electron microscopy, the chorion, serosal cuticle, and amnio‐serosal cuticle of the fixed embryos were carefully removed using forceps in 70% ethanol. The embryos were then dehydrated through a graded ethanol series, transferred to t‐butyl alcohol, and dried using a t‐butyl freeze drier (VFD‐21S, Vacuum Device Inc., Ibaraki). After coating with gold, the embryos were observed using a TM‐1000 Miniscope (Hitachi High‐Technologies Co., Tokyo).

For section observation, the fixed and stored eggs or embryos were rehydrated through a graded ethanol series and then transferred to a 3% hot agar solution on a slide glass, where they were carefully oriented. The embryos were embedded in paraffin (Paraplast X‐TRA, Fisher HealthCare) and sectioned into 5 μm thick slices. To soften the chorion, some eggs were immersed in a mixture of 70% ethanol and 50% ammonium mercaptoacetate in a 9:1 ratio for 24 h before paraffin infiltration, following the method of Tanizawa et al. (2007). The sections were stained with Mayer's acid hemalum and eosin (Eosin Yellowish, Nacalai), and in some cases, supplementary staining was performed using a 0.01% fast green FCF (Tokyo Chemical Industry Co.) alcoholic solution, as described by Tanizawa et al. (2007).

The fixed eggs were stained with a 0.1% DAPI/PBS solution for 12 h and then observed using a fluorescence stereomicroscope (MZ 10 F, Leica Microsystems, Tokyo) under UV light.

## 3 Results

The egg period of *A. japonicus* was approximately 20 days at a temperature of 20°C ± 0.5°C. The embryonic development of *A. japonicus* was divided into 10 stages, following the classification system of Tanaka (2001).

### 3.1 Eggs

**Figure 2:**
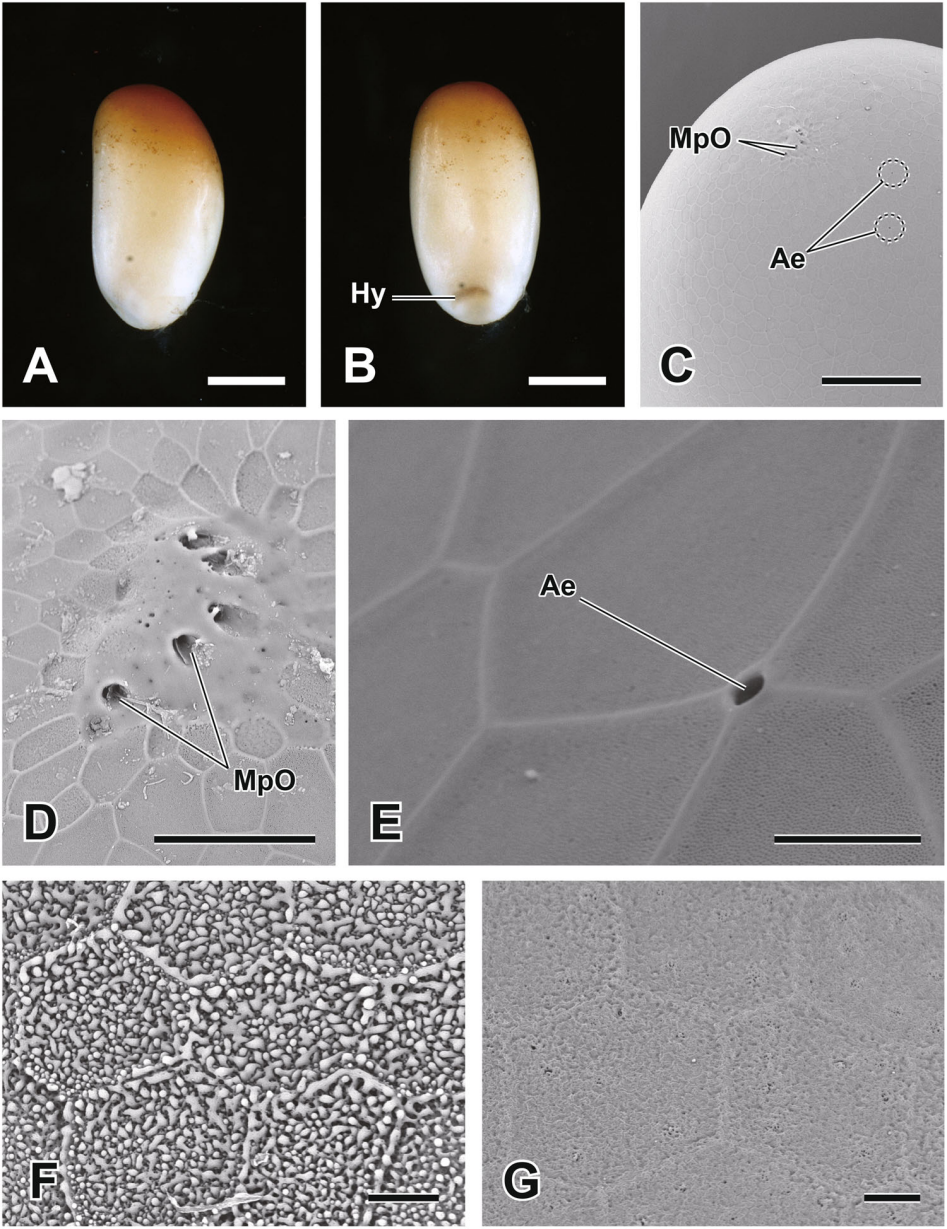
An egg of the giant water bug *Appasus japonicus* and its ultrastructure. Lateral view of newly laid egg (Stage 1: meiotic maturation, fertilization, and cleavage) (A) and dorsal view (B). The micropyles are located at the anterior pole of the egg (C, D), and multiple aeropyles are also observed (C, E). The hydropyle at the posterior pole exhibits a surface structure (F) distinct from other regions of the chorion (G). Scale bars in A, B = 500 μm; in C = 200 μm; in D = 50 μm; in E, F, G = 10 μm. Ae, aeropyle; Hy, hydropyle; MpO, micropylar opening.

The eggs of *A. japonicus* are elliptical, measuring approximately 1.8 mm in length and 0.8 mm in width. The anterior region of the egg is brownish, while the rest remains milky white (Figure 2A,B). At the anterior pole, approximately five micropyles are arranged, each with a micropylar opening of about 5 μm (Figure 2C,D). Aeropyles are distributed across the chorion, with each aeropylar opening measuring approximately 2 μm (Figure 2C,E). The hydropyle is located in the posterior region of the dorsal side of the egg (Figure 2B). This structure facilitates water exchange, and its surface morphology differs significantly from the rest of the chorion (Figure 2F,G). As previously reported by Tanaka (2001), egg size increases from Stage 7 onward, reaching a final length of approximately 2.4 mm and a width of 1.0 mm.

### 3.2 Stage 1‐2

**Figure 3:**
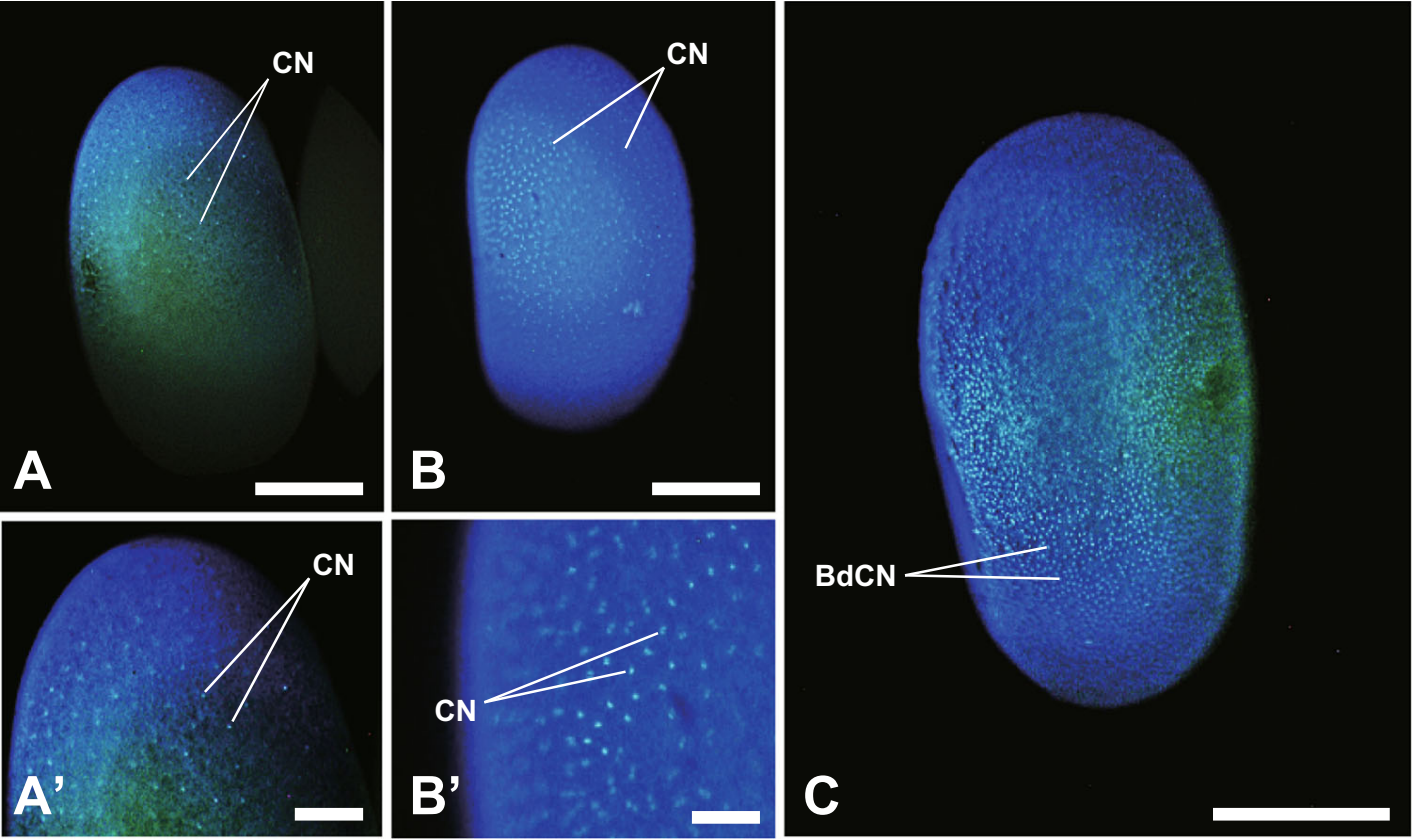
Embryonic development of *Appasus japonicus* (Stages 2–3: Cleavage to Blastoderm Formation). Cleavage nuclei migrate to the egg surface and form the blastoderm. Scale bars in A, B, C = 500 μm; in A’ = 200 μm; in B’ = 100 μm. BdCN, blastoderm cell nucleus; CN, cleavage nucleus.

In this study, we were unable to observe the detailed early embryonic development; however, egg cleavage was identified as the superficial type, as in most insects (Figure 3A,A’).

### 3.3 Stage 3

Cleavage nuclei at the egg periphery, on dividing a few more times, form a unicellular layer, or the blastoderm (Figure 3B,B’).

### 3.4 Stage 4

**Figure 4:**
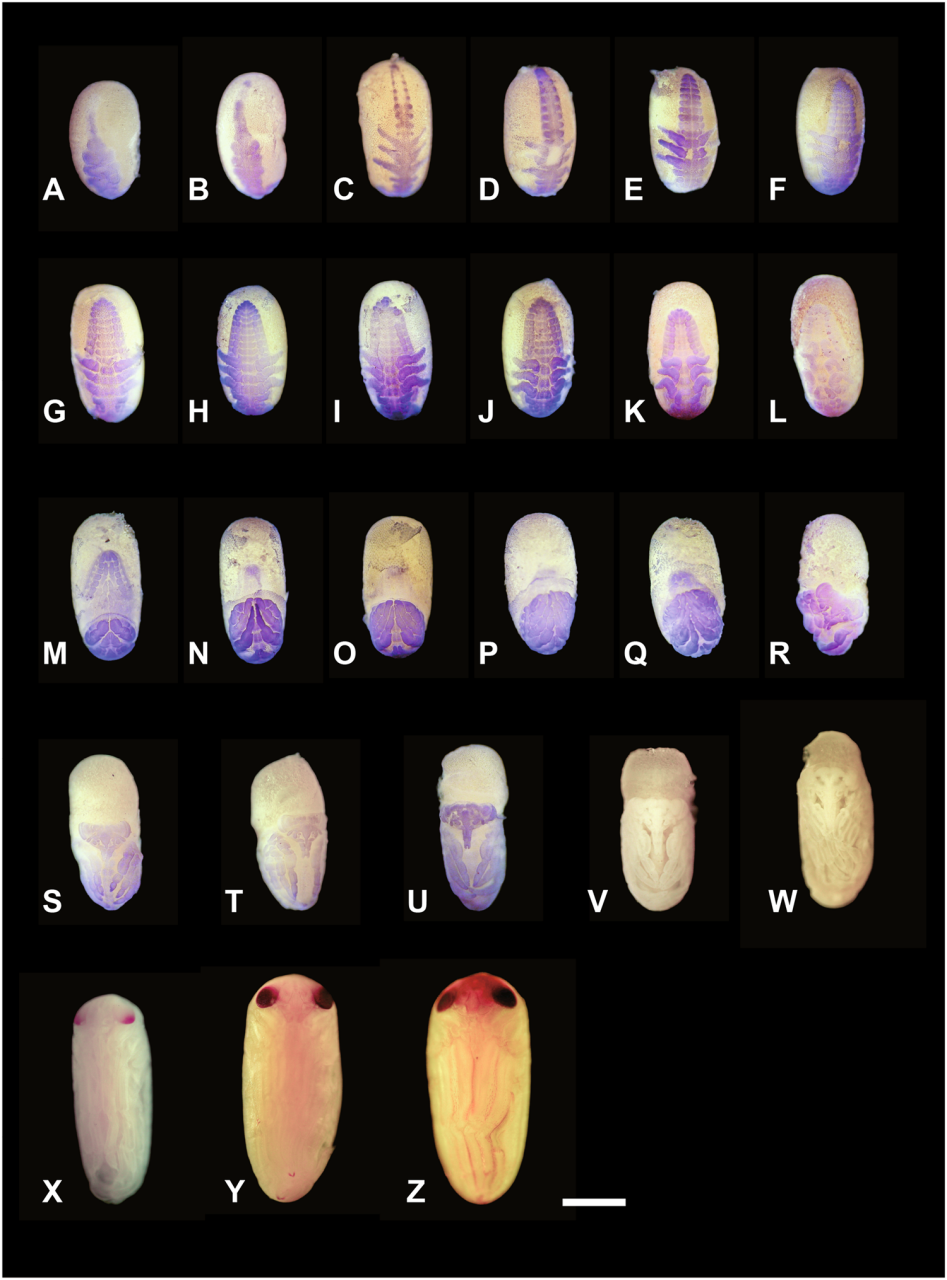
The embryonic development process of *Appasus japonicus*. The stage of embryonic development from the germ band formation to the beginning of segmentation and up to hatching. Due to make it easier to observe the embryos, the chorion covering the surface of each egg was peeled off and removed with tissue forceps, subsequently these embryos were fixed with alcoholic Buin's fluid. Stages 4–7 (A–U) embryos were stained with Mayer's acid hemalum, which specifically stains cell nuclei. Stages 8–10 (V–Z) embryos dissected without any staining. Embryos were photographed under a stereomicroscope. Scale bar = 500 μm.

**Figure 5:**
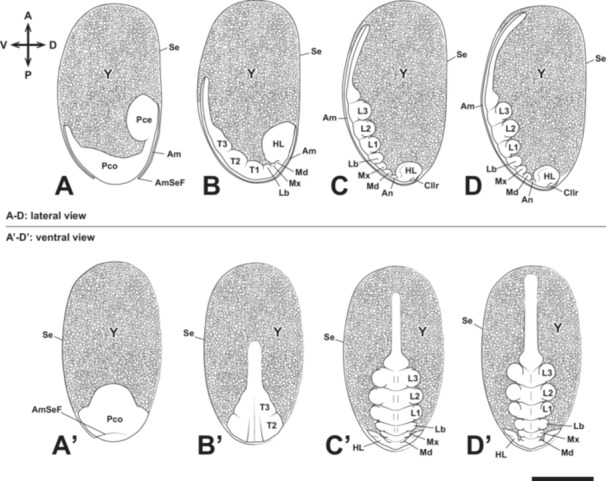
Embryonic development of *Appasus japonicus* (Stage 4: germ band formation and elongation, and appendage rudiment formation). The head gradually moves toward the posterior pole of the egg, while the abdomen elongates toward the anterior pole. Segmentation begins in the head, gnathal, and thoracic segments, whereas the abdominal segments undergo segmentation after Stage 5. Scale bar = 500 μm. A–D, lateral view; A’–D’, ventral view. Am, amnion; AmSeF, amnio‐serosal fold; An, antenna; Cllr, clypeolabrum; HL, head lobe; L1‐3, pro‐, meso‐ and metathoracic legs; Lb, labium; Md, mandible; Mx, maxilla; Pce, protocephalon; Pco, protocorm; Se, serosa; T1‐3, pro‐, meso‐ and metathoracic segments; Y, yolk.

The germ band forms in the posterior region of the egg (Figure 3C). After this stage, the embryos could be observed using a stereomicroscope (Figure 4). The protocephalon develops in the posterior half of the egg. At the same time, the protocorm differentiates at the posterior pole (Figure 5A). The protocephalon is heart‐shaped (viewed from the dorsal side), with a slightly narrower boundary region between it and the protocorm (Figure 5A). Additionally, the amnio‐serosal fold is observed at the posterior pole of the egg (Figure 5A).

The protocephalon shifts toward the posterior pole of the egg, the protocorm moves ventrally and elongates along the ventral surface, with its caudal end positioned near the middle of the egg (Figures 4A, and 5B). As the protocorm extends further toward the anterior pole, the head lobe, gnathal segments, thoracic, and abdominal regions become distinguishable. The amnio‐serosal fold fuses, forming the amniotic cavity within the embryo, and the serosa subsequently encloses the entire egg surface. The width of the abdominal region is approximately one‐third that of the thoracic region (Figure 5B).

The protocephalon remains at the posterior pole of the egg until the onset of katatrepsis (Figures 4B, and 5C,D). Meanwhile, the protocorm continues to elongate, eventually reaching the anterior pole (Figures 4B, and 5C,D). During this stage, the caudal end of the protocorm slightly invaginates into the yolk.

Paired clypeolabral rudiments and paired antennal, gnathal (mandibular, maxillary, and labial), and thoracic appendage rudiments are differentiated (Figure 5B). Among these, the thoracic appendage rudiments are the largest, followed in descending order by the labial, maxillary, mandibular, and antennal rudiments (Figure 5C). The prothoracic, mesothoracic, and metathoracic appendage rudiments are nearly equal in size (Figure 5D).

**Figure 6:**
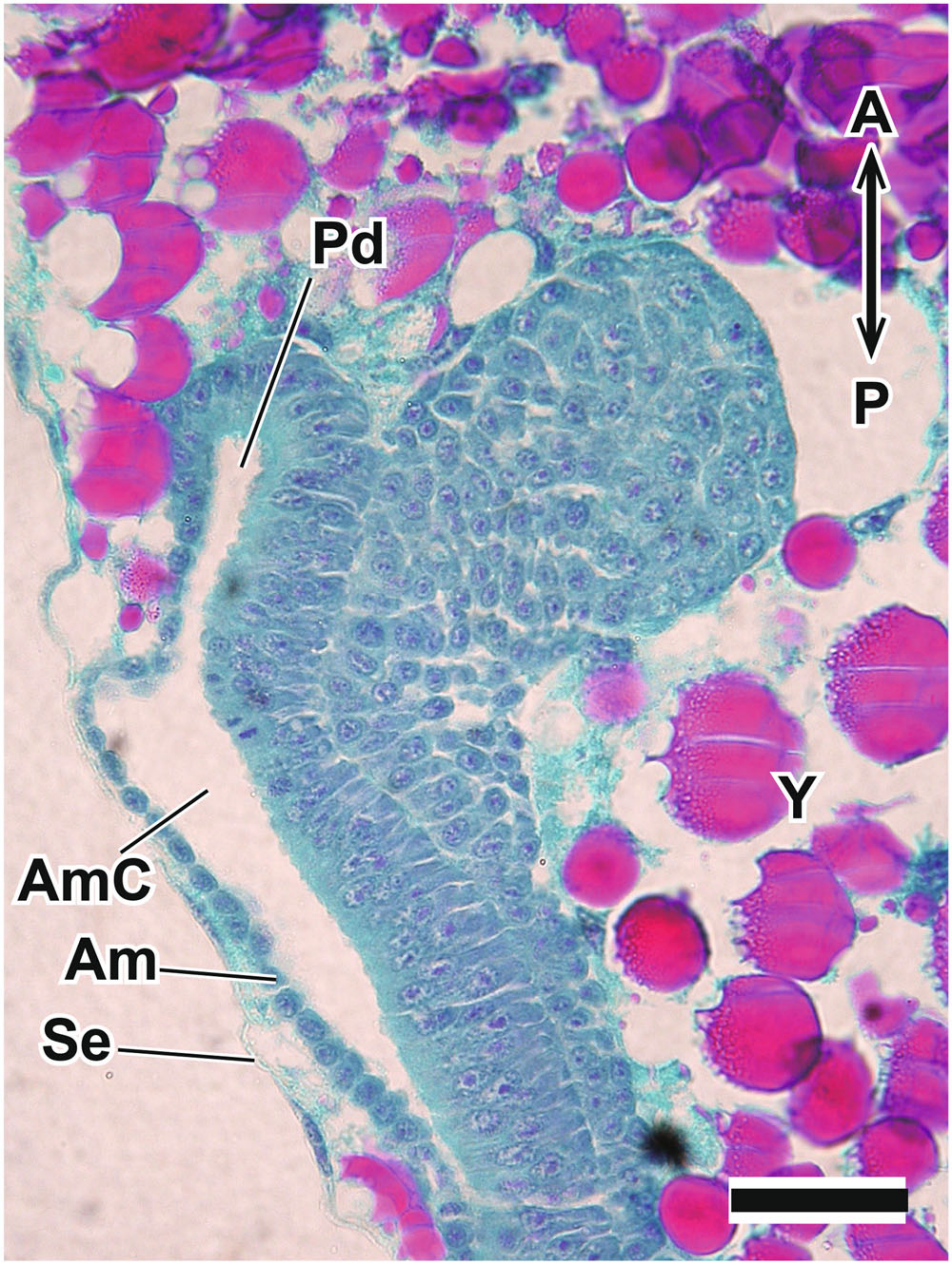
Sagittal section of the posterior region of the embryo of *Appasus japonicus* at Stage 4. Scale bar = 50 μm. Am, amnion; AmC, amniotic cavity; Pd, proctodaeum; Se, serosa; Y, yolk.

At this stage, the stomodaeum forms the posterior region of the clypeolabral rudiments (Figure 5C), and the proctodaeum develops at the caudal end of the abdomen (Figure 6).

### 3.5 Stage 5

**Figure 7:**
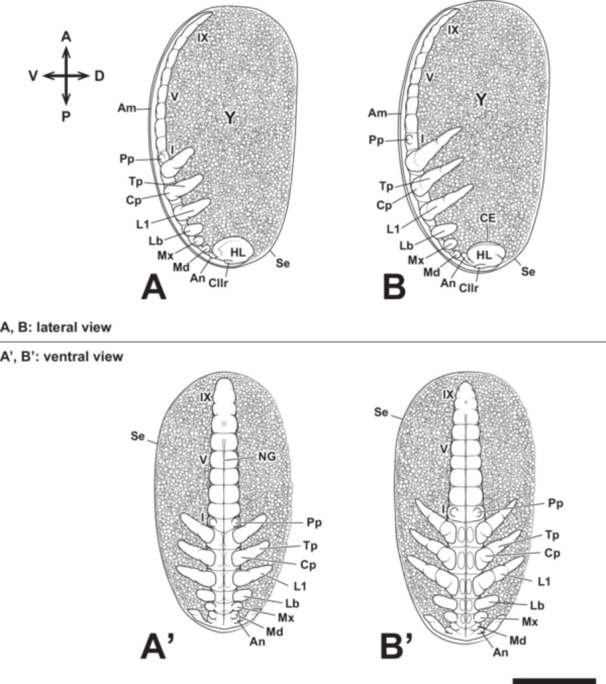
Embryonic development of *Appasus japonicus* (Stage 5: elongation and segmentation of appendage rudiments, and segmentation of abdominal segments). At Stage 5, abdominal segmentation begins, and structures such as abdominal swellings and dorsal plates are formed. Scale bar = 500 μm. A, B, lateral view; A’, B’, ventral view. I, V, IX, first, fifth and ninth abdominal segments; Am, amnion; An, antenna; CE, compound eye; Cllr, clypeolabrum; Cp, coxopodite; HL, head lobe; L1, prothoracic legs; Lb, labium; Md, mandible; Mx, maxilla; NG, neural groove; Pp, pleuropodium; Se, serosa; Tp, telopodite; Y, yolk.

At this stage, segmentation begins in the abdominal region (Figures 4C,D and 7A,B), starting from the anterior abdominal segments and progressing toward the posterior. Ganglionic swellings also form in each segment, beginning from the head and extending into the abdomen. Following this, the neural groove becomes clearly visible (Figures 4C, and 7A’). The ganglionic swellings are more pronounced in the gnathal and thoracic segments than in the abdominal segments, and the neural groove is more clearly observed in the anterior abdominal region than in the posterior (Figures 4C,D and 7A,B). After abdominal segmentation begins, a pair of pleuropodia forms in the first abdominal segment (Figures 4C and 7A). The pleuropodia develop outside the ganglionic swellings, similar to the formation of gnathal and thoracic appendages. Segmentation of the abdomen is completed during this stage, resulting in 11 segments. The thoracic appendages elongate and are subdivided into two segments: the coxopodite and the telopodite. During this stage, the telopodite of the thoracic appendages further divides, and the antennae also undergo segmentation; however, the maxilla and labium remain undivided. Segmentation does not occur in the mandible, as observed in other insects.

The serosal cuticle begins to separate from the serosa, which covers the entire surface of the egg in the previous stage. The serosal cuticle subsequently covers the whole surface of the egg until hatching.

### 3.6 Stage 6

**Figure 8:**
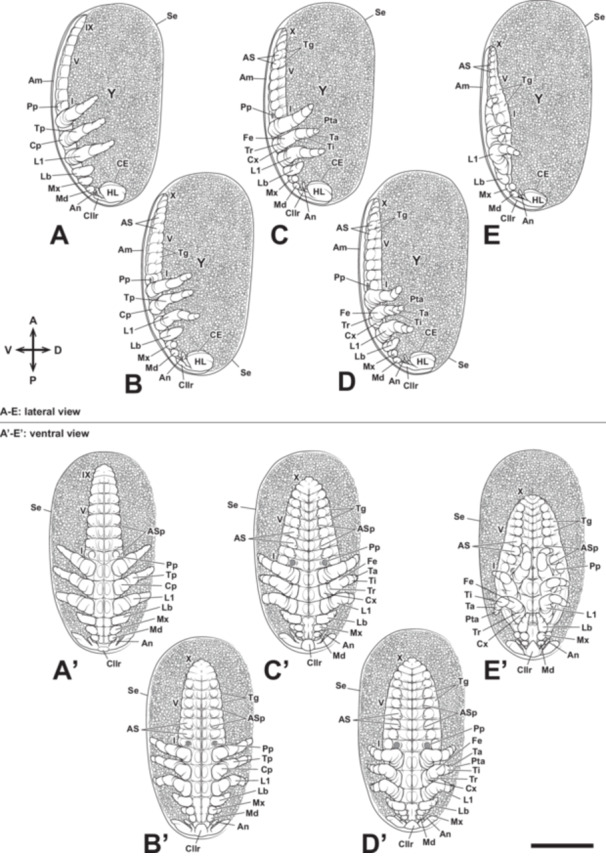
Embryonic development of *Appasus japonicus* (Stage 6: flexion of thoracic appendages). In the latter part of Stage 6, the thoracic appendages are folded inward. Scale bar = 500 μm. A–E, lateral view; A’–E’, ventral view. I, V, IX, X, first, fifth, ninth and tenth abdominal segments; Am, amnion; An, antenna; AS, abdominal swellings; ASp, abdominal spiracles; CE, compound eye; Cllr, clypeolabrum; Cp, coxopodite; Cx, coxa; Fe, femur; HL, head lobe; L1, prothoracic leg; Lb, labium; Md, mandible; Mx, maxilla; Pp, pleuropodium; Pta, pretarsus; Se, serosa; Ta, tarsus; Tg, tergite; Ti, tibia; Tp, telopodite; Tr, trochanter; Y, yolk.

After abdominal segmentation is complete, the width of the abdomen begins to increase while its length decreases (Figures 4E,F and 8A,B). Paired abdominal swellings form in the 1st through 8th abdominal segments. Subsequently, the pleuropodia collapse into the body (Figures 4F and 8B). The abdominal swellings become clearly visible as this stage progresses, forming the ganglionic swellings and terga. The abdominal swellings in segments 2–8 are positioned serially relative to the pleuropodia, while the abdominal swelling in the 1st abdominal segment is positioned outward from the pleuropodia. As the abdominal swellings further develop, the sternum begins to form between the ganglionic swellings and the abdominal swellings (Figures 4E,F and 8A,B), with the spiracles forming in the anterior region of the abdominal swellings (Figures 4E and 8A). The width of the abdomen continues to increase as its length decreases, and the abdominal swellings further develop. The segmental boundary between the 10th and 11th abdominal segments gradually becomes less distinct. In belostomatid species, spiracles are formed in the abdomen as well as in the mesothoracic and metathoracic segments (Miller 1961; Parsons 1972); however, the process of thoracic spiracle formation was not observed in this study.

At this stage, the maxilla and labium elongate and divide into the coxopodite and telopodite (Figures 4E–G and 8A–C). Subsequently, the telopodite of the labium divides into two segments, and both the labium and thoracic appendages begin to fold the ventral area of the embryo (Figures 4E–G and 8A–C). The coxopodites of the thoracic appendages divide into two segments, the coxa and subcoxa, while the telopodites of the thoracic appendages divide into five segments: the trochanter, femur, tibia, tarsus, and pretarsus (Figures 4H–K and 8C,D). After the thoracic appendages fold the ventral area of the embryo, they extend to cover the surface of the abdomen (Figures 4L and 8E).

### 3.7 Stage 7

**Figure 9:**
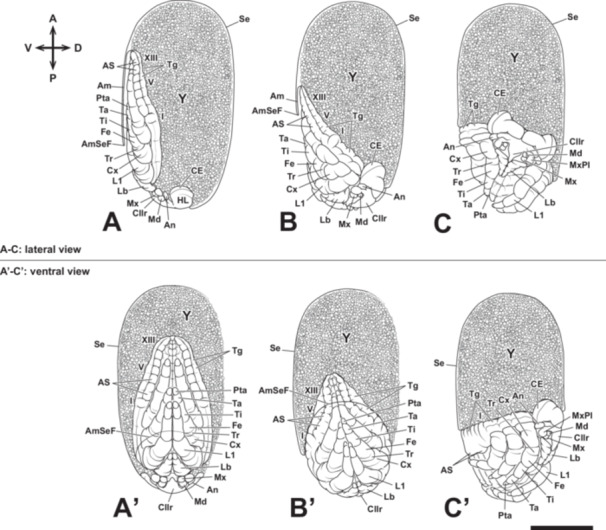
Embryonic development of *Appasus japonicus* (Stage 7: embryo reorientation). At Stage 7, katatrepsis occurs due to the dissolution of the amnio‐serosal fold. Simultaneously, a reorientation movement involving rotation occurs. Scale bar = 500 μm. A–C, lateral view; A’–C’, ventral view. I, V, VIII, first, fifth and eighth abdominal segments; Am, amnion; AmSeF, amnio‐serosal fold; An, antenna; AS, abdominal swellings; CE, compound eye; Cllr, clypeolabrum; Cx, coxa; Fe, femur; HL, head lobe; L1, prothoracic leg; Lb, labium; Md, mandible; Mx, maxilla; MxPl, maxillary plate; Pp, pleuropodium; Pta, pretarsus; Se, serosa; Ta, tarsus; Tg, tergite; Ti, tibia; Tr, trochanter; Y, yolk.

**Figure 10:**
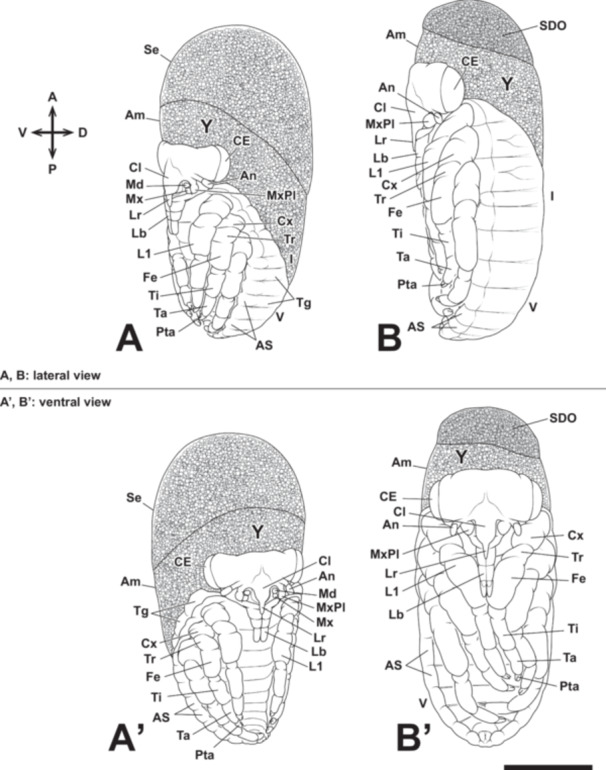
Embryonic development of *Appasus japonicus* (Stage 8: rapid embryonic growth, compound eye pigmentation, and dorsal closure). At Stage 8, katatrepsis is completed, and the embryo undergoes a rapid increase in length. Scale bar = 500 μm. A, B, lateral view; A’, B’, ventral view. I, V, first and fifth abdominal segments; Am, amnion; An, antenna; AS, abdominal swellings; CE, compound eye; Cl, clypeus; Cx, coxa; Fe, femur; L1, prothoracic leg; Lb, labium; Lr, labrum; Md, mandible; Mx, maxilla; MxPl, maxillary plate; Pta, pretarsus; Se, serosa; SDO, secondary dosal organ; Ta, tarsus; Tg, tergite; Ti, tibia; Tr, trochanter; Y, yolk.

The left and right labium contact at the ventral median line of the embryo, and the telopodite of the labium is divided into three segments. The amnio‐serosal fold ruptures, and katatrepsis occurs (Figures 4, 9, and 10A), marking the completion of provisional dorsal closure. In the early katatrepsis stage, the eyes show a slight red coloration. The embryo undergoes a 180° rotation along the anteroposterior axis of the egg during katatrepsis in the embryonic development of *A. japonicus* (Figures 4, 9, and 10A). This rotation is completed during katatrepsis, and all observed embryos rotated according to the right‐hand rule. After katatrepsis is complete, the borders of the 9th–11th abdominal segments become difficult to observe. Katatrepsis is considered complete when the anterior edge of the embryo reaches the midpoint of the egg and the posterior edge reaches the posterior region of the egg. Following katatrepsis, the ventral surface of the abdomen becomes temporarily visible as the thoracic appendages, which previously covered the abdomen, are opened. The embryo appears to be shouldering the yolk on its back, with the abdominal swellings arranged along the edge of the abdomen. The tergum elongates from the edge of the embryo to the ventral median line, initiating definitive dorsal closure.

### 3.8 Stage 8

**Figure 11:**
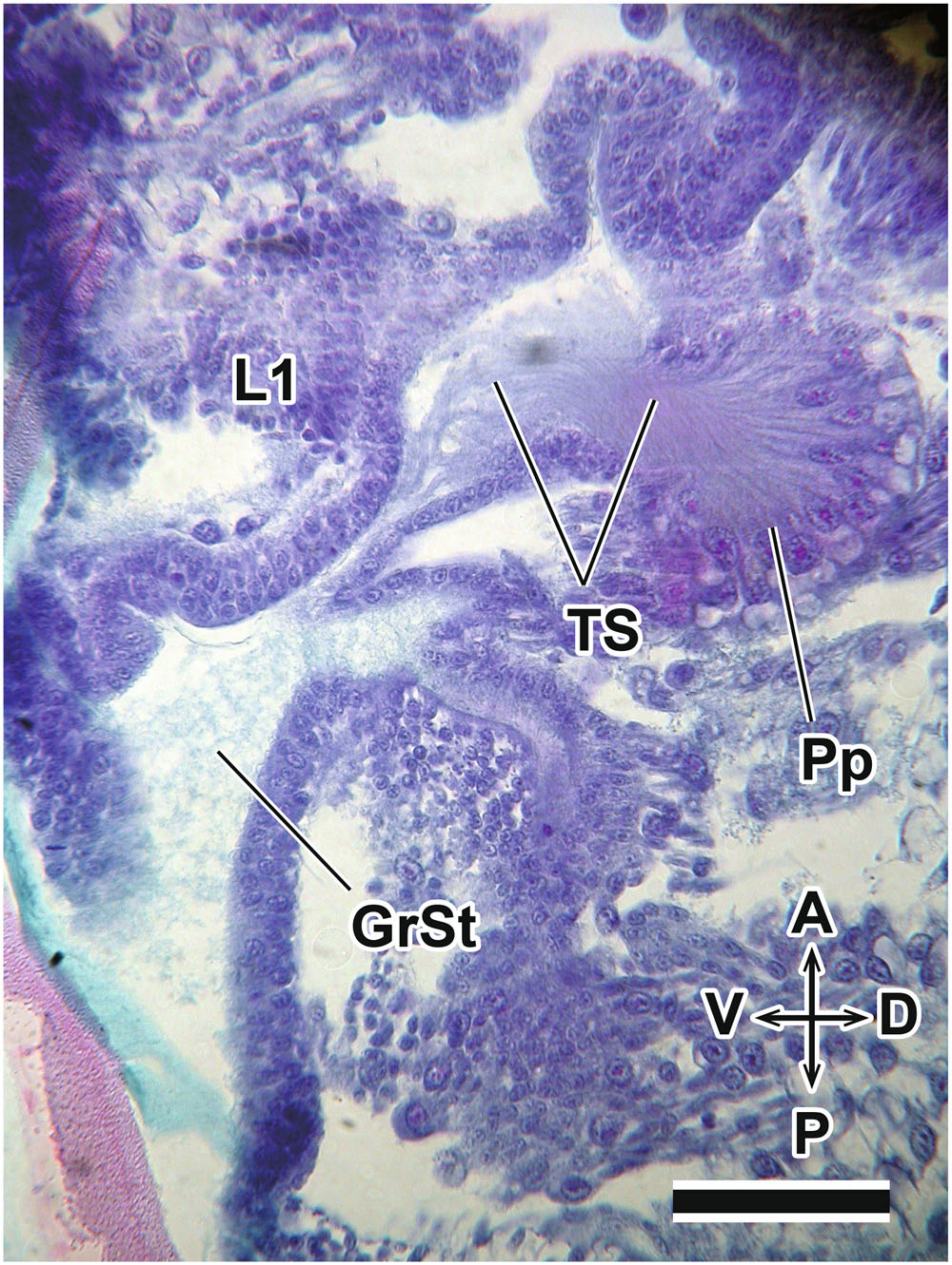
*Appasus japonicus*, sagittal section of the pleuropodium at Stage 8. Scale bar = 50 μm. GrSt, granular structure; L1, prothoracic leg; Pp, pleuropodium; TS, thread‐like structure.

After katatrepsis is completed at the end of Stage 7, definitive dorsal closure begins with the elongation of the tergum edge. Dorsal closure, driven by the elongation of the tergum, is completed during this stage. The definitive dorsal closure proceeds from the posterior part of the abdomen toward the anterior part of the embryo. During this process, the embryo elongates, and the head gradually shifts to the anterior part of the egg (Figures 4S–W and 10A,B). The thoracic appendages also elongate simultaneously, with the left and right legs moving toward the midline of the abdomen to cover the ventral region. Additionally, the pretarsus of each thoracic appendage differentiates into claws, and the pair of metathoracic legs continue to elongate, overlapping each other (Figures 4W and 10B’). At this stage, the abdominal swellings that had formed along the ventral edge of the embryo become flattened (Figures 4S–W and 10A,B). The telopodite of the maxilla differentiates into the maxillary plate, which covers the mandible and the coxopodite of the maxilla. Moreover, the pleuropodia secrete enzymes during this stage (Figure 11).

After dorsal closure of the abdominal region is completed, dorsal closure of the thoracic region begins. At this stage, the yolk remains in the dorsal part of the prothorax. Dorsal closure proceeds by covering the remaining yolk, and the secondary dorsal organ, which originates from the retracted serosa (once its role in cuticle secretion is finished), becomes prominent in the dorsal region of the head‐thorax area (Figure 10B). As dorsal closure progresses, the head shifts further toward the anterior part of the egg, and most of the yolk is covered by the embryo. The color of the eyes gradually darkens.

### 3.9 Stage 9

**Figure 12:**
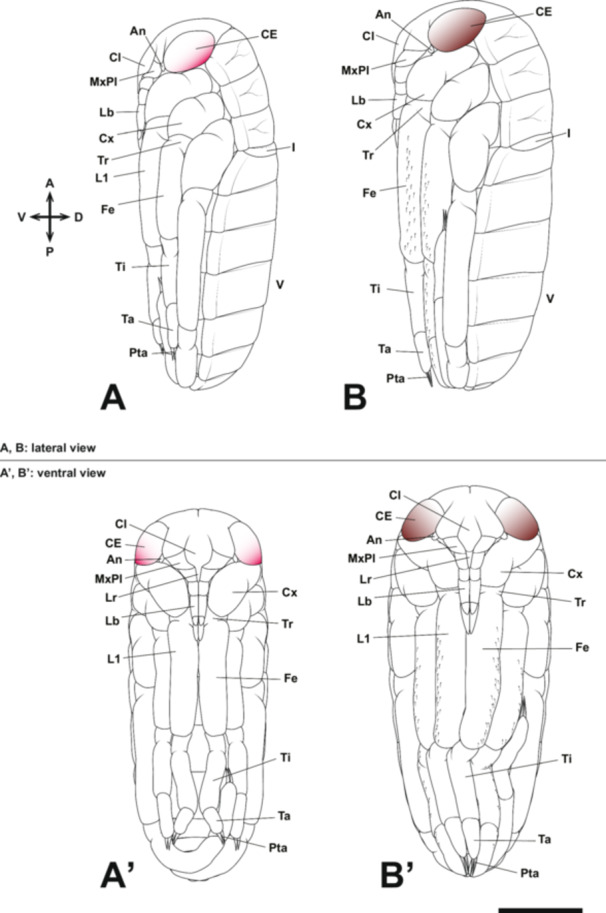
Embryonic development of *Appasus japonicus* (Stage 9: further increase in embryo size). At Stage 9, the embryo increases in size, and the pigmentation of the claws and compound eyes becomes more pronounced. Scale bar = 500 μm. A, B, lateral view; A’, B’, ventral view. I, V, first and fifth abdominal segments; An, antenna; CE, compound eye; Cl, clypeus; Cx, coxa; Fe, femur; L1, prothoracic leg; Lb, labium; Lr, labrum; MxPl, maxillary plate; Pta, pretarsus; Ta, tarsus; Ti, tibia; Tr, trochanter.

The secondary dorsal organ becomes completely covered by the embryo and is eventually incorporated into the gut, making it no longer visible externally. The maxillary plate expands, and the gap between the maxillary plate and the first segment of the labium is completely filled, as is the gap between the maxillary plate and the clypeus. The embryo continues to elongate, and the colored area of the compound eyes expands (Figures 4X and 12A). The thoracic appendages also elongate further, particularly the femur and tibia of the metathoracic appendage (hind leg). The tibia of the metathoracic appendage curves at the posterior pole of the egg, while its tarsus and pretarsus elongate further along the lateral side of the embryo toward the anterior pole of the egg (Figures 4X and 12A). During this stage, the width of the embryo begins to increase, and its morphology gradually approaches that of the first instar nymph. Notably, egg teeth, which are commonly observed in many insects for breaking the chorion, are not present in *A. japonicus*.

The embryo develops a faint brownish color, and the claws also become pigmented during this stage. The color of the compound eyes shifts from red to a blackish red, and the compound eyes become visible through the chorion (Figures 4Y,Z and 12B). The setae also become pigmented and are clearly visible at this stage (Figures 4Y,Z and 12B). After completing all the stages, the embryonic development concludes, and the first instar nymph hatches.

### 3.10 Stage 10 (First Nymphs)

The eggs hatch on the males’ back, and the hatch proceeds from the egg, which is oviposited earlier. Firstly, the anterior region of the chorion breaks horizontally direction except for part of the dorsal region, and the head of the first instar nymph gradually comes out. After that, the first instar nymph bends its body backward and spreads out its thoracic appendages when half of the thoracic appendages come out, and the first instar nymph bends its body forward. The nymph keeps this pose, and then the male, who carries eggs, enters the water, and the nymph completely comes out from the chorion.

After hatching, the cuticle of nymph expands, and body size becomes a little bit large. The body color of hatched nymph is light yellow and compound eyes’ color are blackish red. Because the yolk is included in the midgut when the dorsal closure completed, the color of midgut is observed light green. After few minutes from hatching, the nymph's body cuticle hardens, and the body color changes to mainly brownish with some light yellowish spots.

**Figure 13:**
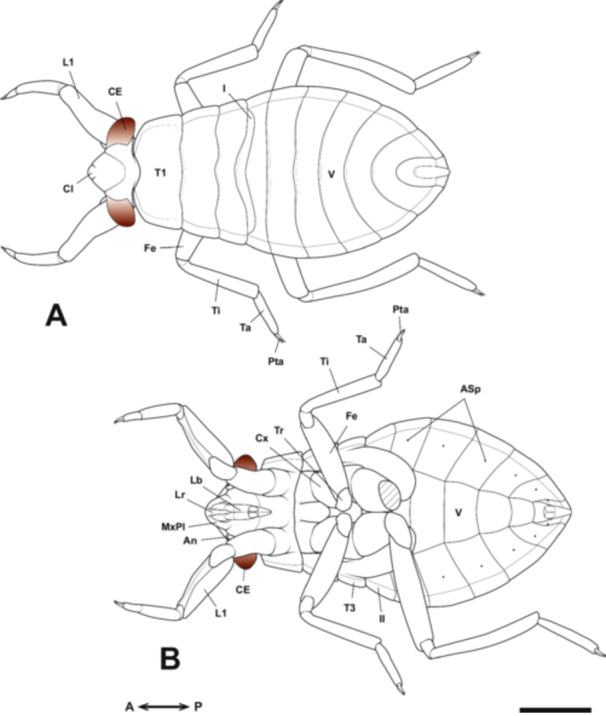
Embryonic development of *Appasus japonicus* (Stage 10: first instar nymph). Dorsal view (A). Ventral view, with the left hind leg removed to visualize the ventral abdominal structures (B). Scale bar = 1 mm. I, II, V, first, second and fifth abdominal segments; An, antenna; ASp, abdominal spiracles; CE, compound eye; Cl, clypeus; Cx, coxa; Fe, femur; L1, prothoracic leg; Lb, labium; Lr, labrum; MxPl, maxillary plate; Pta, pretarsus; Ta, tarsus; T1, 3, pro‐ and metathoracic segments; Ti, tibia; Tr, trochanter.

The prothoracic segment is largest and the metathoracic segment is smallest in thoracic segments (Figure 13A). While the abdominal segments start to differentiate in Stage 5, the length of each segment to be narrow. After this stage, the boundaries between the 9th to 11th abdominal segments become indistinct, making it appear like only eight abdominal segments when observed from the ventral side (Figure 13B). In belostomatids, the spiracles of the abdominal segments are not externally visible in the 1st and 2nd abdominal segments because their openings are located deep within the grooves between the thoracic and abdominal segments. However, the spiracles of the 3rd to 8th abdominal segments are exposed, making their openings observable (Figure 13B).

### 3.11 Mouthparts’ Development of *A. japonicus*

**Figure 14:**
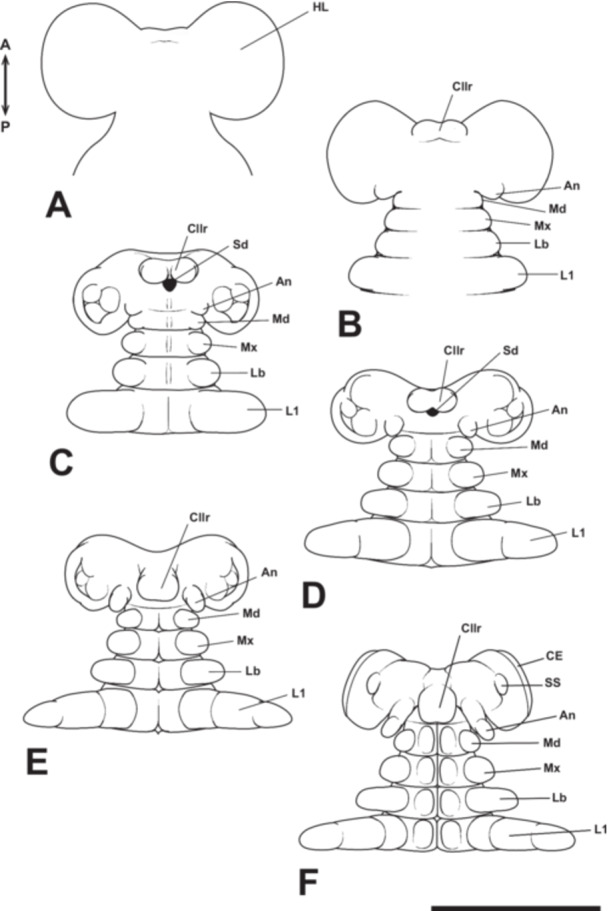
Mouthpart formation of *Appasus japonicus* (Stage 4: A–C and Stage 5: D–F). Scale bar = 500 μm. An, antenna; CE, compound eye; Cllr, clypeolabrum; HL, head lobe; L1, prothoracic leg; Lb, labium; Md, mandible; Mx, maxilla; Sd, stomodaeum; SS, small swellings.

At Stage 4, the germ band is formed, followed by segmentation progressing from its anterior region. In the head, segmentation occurs in the antennal segment, intercalary segment, mandibular segment, maxillary segment, and labial segment. Among these, some segments also exhibit appendage differentiation, leading to the formation of the antennal rudiment, mandibular rudiment, maxillary rudiment, and labial rudiment (Figure 14A–C). Anterior to the antennae, the differentiation of the clypeolabral rudiment is observed. However, based on external morphology, it remains unclear whether this structure represents an appendage and to which segment it belongs. The segmentation pattern anterior to the antennal segment is not well‐defined, and the boundary of the intercalary segment, which lacks differentiated appendage‐like structures, is somewhat indistinct. During its early formation, the clypeolabral rudiment appears as a bilobed structure. In the latter half of Stage 4, as a neural groove becomes visible along the ventral midline, the stomodaeum forms posterior to the clypeolabral rudiment (Figure 14C).

At Stage 5, the clypeolabral rudiment extends posteriorly along the body axis, gradually covering the stomodaeum. The initially bilobed structure transforms into a single, larger mass‐like clypeolabral rudiment (Figure 14D–F). As development progresses, the antennal rudiment elongates and is subdivided into two segments, the coxopodite and telopodite, following a delay compared to the segmentation of the thoracic appendages (Figure 14F). At this stage, the rudiments of the compound eyes form, and a pair of small swellings appear on the head (Figure 14F).

**Figure 15:**
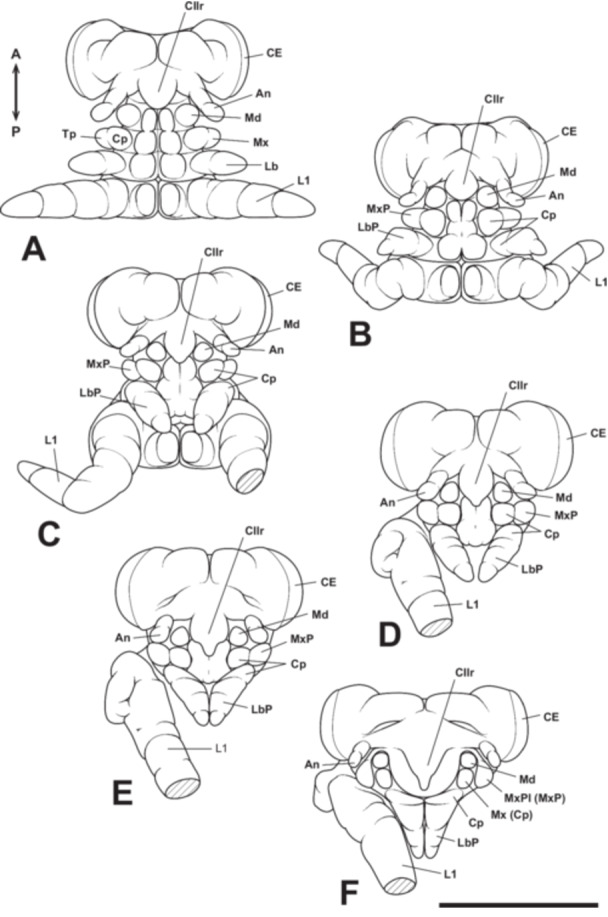
Mouthpart formation of *Appasus japonicus* (Stage 6: A–C and Stage 7: D–F). Scale bar = 500 μm. An, antenna; CE, compound eye; Cllr, clypeolabrum; Cp, coxopodite; L1, prothoracic leg; Lb, labium; LbP, labial palp; Md, mandible; Mx, maxilla; MxP, maxillary palp; MxPl, maxillary plate; Tp, telopodite.

Further development leads to Stage 6, during which the maxillae and labium elongate laterally. Around this stage, these rudiments also undergo segmentation, forming two distinct subdivisions corresponding to the coxopodite and telopodite (Figure 15A). Additionally, the small swellings observed on the head in the previous stage disappear (Figure 15A). At this stage, the clypeolabrum extends beyond the antennal segment and reaches the mandibular segment, rendering the intercalary segment no longer distinguishable (Figure 15B). The antennae also extend as far as the mandibular segment, resulting in the mandibles being positioned between the antennae and the clypeolabrum (Figure 15B). Around this time, the telopodite region of the labium undergoes further segmentation, forming three segments. Additionally, the mandibular, maxillary, and labial segments become compressed, leading to the fusion of their respective neural ridges. As development progresses further, the labium folds inward (toward the midline) before the thoracic appendages undergo similar movements (Figure 15C).

At Stage 7, the left and right labium come into contact along the midline, and the telopodite region of the labium undergoes further segmentation, forming third telopodal segments (Figure 15D,E). The clypeolabrum tapers at its distal end, initiating its differentiation into the proximal clypeus and the labrum. As development progresses, the labrum extends further posteriorly (Figure 15D–F). By the end of Stage 7, the left and right labial lobes, now closely apposed, develop pointed distal ends, and all four segments from the base to the tip make direct contact along the midline (Figure 15F).

**Figure 16:**
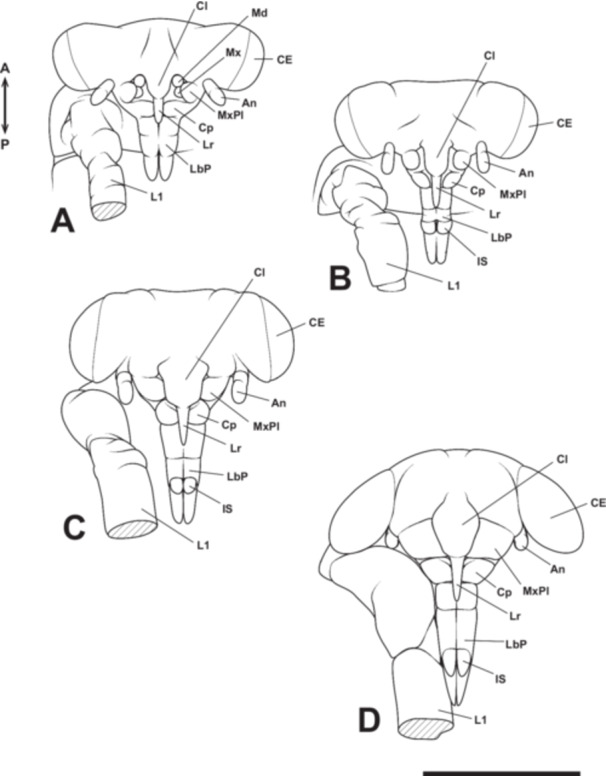
Mouthpart formation of *Appasus japonicus* (Stage 8: A–C and Stage 9: D). Scale bar = 500 μm. An, antenna; CE, compound eye; Cl, clypeus; Cp, coxopodite; IS, intercalary sclerites; L1, prothoracic leg; Lr, labrum; LbP, labial palp; Md, mandible; MxPl, maxillary plate.

At Stage 8, the maxillary telopodite extends to cover the mandibles and maxillary coxopodite, forming a structure known as the maxillary plate (Figure 16A–C). Consequently, from this stage, it is no longer possible to externally observe the development of the mandibles, maxillary coxopodite, and maxillary telopodite (maxillary plate) within the cranial cavity. During this stage, the labrum elongates further, reaching approximately the first telopodal segment of the labium (Figure 16A). Additionally, the ventral surfaces of the left and right labial lobes begin to fuse, and a small groove forms along the midline of the dorsal surface of the labium. This groove persists after hatching and later develops into a sheath‐like structure that houses the mandibular and maxillary stylets within the tubular labrum (i.e., the mandibular and maxillary stylets pass through the tube‐like labrum and are housed within the labium; Figure 1B). A groove also forms in the coxopodal segment of the labium, allowing the labrum to pass through. Furthermore, small intercalary sclerites develop on the second telopodal segment of each labium (Figure 16B). The small intercalary sclerites differentiate from the second telopodal segment of the labium after blastokinesis and form rigid cuticular structures that fit tightly against the third telopodal segment of the labium (Figure 16B–D).

At Stage 9, the maxillary plate completely covers the area above the base of the coxopodal segment of the labium, leaving no gap between it and the clypeus (Figure 16D). Additionally, the intercalary sclerites on the second telopodal segments of the labium become more sharply defined, marking the near completion of mouthpart formation in *A. japonicus* (Figure 16D). Furthermore, since the antennae develop on the ventral side of the head, they become concealed beneath it as the dorsal closure of the head is completed, leaving only their distal ends visible (Figure 16D).

**Figure 17:**
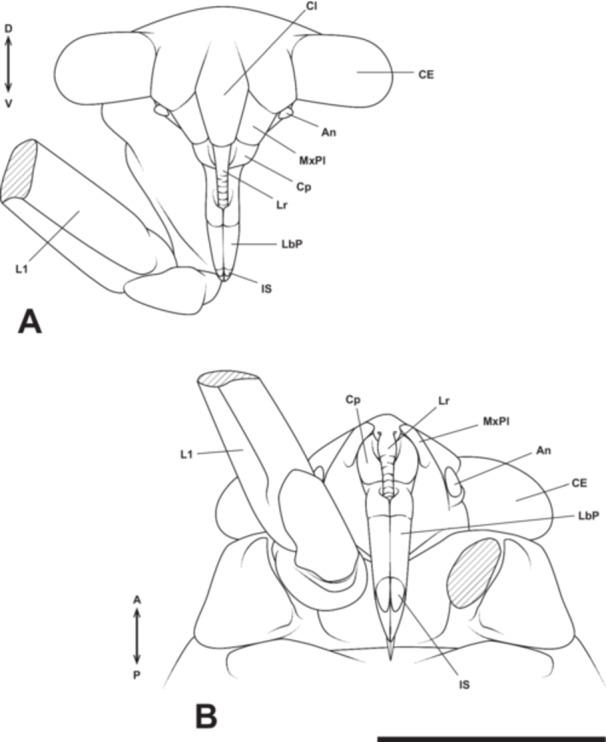
Mouthpart formation of *Appasus japonicus* (Stage 10: first instar nymph). Scale bar = 500 μm. An, antenna; CE, compound eye; Cl, clypeus; Cp, coxopodite; IS, intercalary sclerites; L1, prothoracic leg; Lr, labrum; LbP, labial palp; MxPl, maxillary plate.

In the hatched first instar nymph (Stage 10), the rostrum exhibits a morphology fundamentally identical to that of the adult (Figure 17A,B). The labrum, extending as a slender tubular structure from the clypeus, enclosed the proximal part of the mandibular and maxillar stylets. The coxopodal segment of the labium features a groove accommodating the labrum, while the second segment forms a sheath‐like structure that envelops the distal portion of the labrum, as well as the mandibular and maxillary stylets (Figure 17A). From the second telopodal segment of the labium, intercalary sclerites differentiate, forming a covering structure over the base of the third (terminal) telopodal segment (Figure 17B). The tip of the third telopodal segment is sharp with a small opening, allowing for the taking in and out of the mandibular and maxillary stylets.

## 4 Discussion

### 4.1 Blastokinesis

During insect embryogenesis, dynamic movements known as blastokinesis, including katatrepsis and rotation, are frequently observed (Panfilio 2008). Among these, katatrepsis is particularly notable, involving a 180° rotation of the embryonic body along the anteroposterior axis of the egg. However, katatrepsis is not an isolated event but rather a consequence of a sequential developmental process: (1) formation of the amnio‐serosal fold during anatrepsis, (2) complete coverage of the egg surface by the serosa, (3) secretion of the serosal cuticle over the entire egg surface, and (4) regression of the functionally redundant amnio‐serosal fold. As the amnio‐serosal fold retracts, the cephalic region of the embryo, which serving as the initiation site of the fold, is drawn toward the anterior pole of the egg. This triggers a secondary shift in embryonic posture, leading to katatrepsis (Dorn 1976; Tojo and Machida 1997; Machida and Ando 1998; Lamer and Dorn 2001; Machida et al. 2002). In the present study, we also observed that the cephalic region, which initiates the amnio‐serosal fold, gradually shifts toward the anterior pole of the egg. This movement is followed by a positional shift of the thoracic and abdominal regions (Figure 9A–C). The postural shift observed during katatrepsis in *A. japonicus* is considered a secondary movement accompanying the regression of the amnio‐serosal fold. Meanwhile, the rotational movement occurring simultaneously with katatrepsis appears to result from two factors: (1) the inversion movement itself, a secondary posture adjustment caused by the retraction of the amnio‐serosal fold, and (2) the removal of structural constraints that previously fixed the embryo's position on the egg surface, allowing free rotation once the amnio‐serosal fold has fully regressed. Such rotational movements are thought to be subject to selective pressures in species where eggs are laid on substrates that physically constrain hatching in certain directions, making a specific orientation necessary for successful emergence. A notable example is observed in water striders (Gerridae) and backswimmers (Notonectidae), where embryos undergo rotation after katatrepsis. This mechanism is thought to have evolved to optimize hatching orientation, ensuring that the egg hatches in the most favorable direction (Cobben 1968). In the southern green stink bug, *Nezara viridula* (Heteroptera: Pentatomidae), it has been reported that the eggs positioned at the periphery of the egg mass undergo dorsoventral axis adjustment, ensuring that their ventral side faces inward. This orientation enables the first instar nymphs to aggregate toward the center of the egg mass upon hatching, facilitating the formation of a flock on the empty eggshells (Lockwood and Story 1986). In the giant water bug, Cobben (1968) suggested the possibility of rotational movement during embryogenesis. However, no prior studies have provided direct evidence of such movement. In the present study, detailed observations of embryonic development in *A. japonicus* clearly demonstrated the occurrence of rotational movement simultaneously with katatrepsis, specifically between Stage 7 and Stage 8.

Smith (1976) reported that in belostomatine giant water bugs, first instar nymphs consistently hatch toward the rear of the male that cares for the eggs. It was suggested that this behavior may have adaptive significance, helping to avoid predation by the male immediately after hatching. In the present study, we confirmed the presence of rotational movement in *A. japonicus*, which could be seen as an adaptation to prevent predation by the male parent, as proposed by Smith (1976). However, a question remains regarding whether, in the unlikely event that hatching occurs toward the front of the male, the male would actively predate his own offspring emerging from the egg mass.

### 4.2 Abdominal Morphogenesis

In *A. japonicus*, abdominal morphogenesis begins after the formation of the germ band at Stage 4, with the elongation of the abdominal primordium. In insect embryogenesis, three main patterns of germ band formation are recognized: (1) the “short germ” type, where only a small protocephalon is formed initially, followed by posterior elongation of the germ band and the differentiation of the segment‐forming zone, leading to the sequential formation of body segments posterior to the mandibles, (2) the “long germ” type, in which a long initial germ band is formed, and body segmentation occurs within the already elongated germ band, without further elongation, and (3) the “semi‐long germ” type, which is an intermediate form between the short and long germ types, where the initial germ band consists of the protocephalon and the mandibular‐thoracic region, and abdominal formation progresses from the elongated region as development proceeds (Ando 1991). Based on the classification of embryonic movement types by Ando (1991) and the observations from this study, it can be concluded that *A. japonicus* follows the semi‐long germ type of germ band formation. After the abdominal elongation in Stage 4, segmentation of the abdomen begins slightly later than the thoracic segmentation in Stage 5 (Figure 7A’,B’). This is similar to the abdominal formation pattern observed in *Oncopeltus fasciatus*, Lygaeidae (Butt 1949). In Stage 6, the abdominal segments differentiate into 11 segments (Figure 8A’), but in Stage 7, a rapid shortening of the abdomen results in the extreme compression of the 9th to 11th segment, while the first abdominal segment progressively narrows in the anteroposterior axis and becomes hidden within the thoracic ventral plate of the third thoracic segment, making it visible only from the dorsal side. Although such abdominal morphogenesis has not been reported in *O. fasciatus*, it is generally accepted that the first abdominal segment is not visible from the external abdominal surface in other hemipteran species (Miller 1961; Parsons 1972; Yasunaga et al. 1993). Similarly, in the basal group of the paraneopteran insects, such as the order Psocoptera, the first abdominal segment has also been reported to be observable only from the dorsal side (Yoshizawa 2005). Therefore, the abdominal morphology in *A. japonicus* follows a basic plan shared by paraneopteran insects, which could provide crucial insights into the ground plan of abdominal structure in these insects. Future studies should focus on determining at which developmental stage and through what evolutionary processes this abdominal formation pattern was acquired.

Arthropods, including insects, are characterized by a fundamentally metameric body plan, where repeated, homologous segments form the basis of their morphology. Building upon this fundamental segmentation plan, arthropods have diversified through a process known as tagmosis, in which multiple segments merge into functionally specialized compartments (Snodgrass 1935). Thus, understanding the segmentation is critical to comprehending the fundamental body plan of arthropods. One of the key criteria for defining a segment includes the possession of a paired ganglion, a paired coelomic cavity, and a pair of appendages (Snodgrass 1935; Tojo and Machida 1997). In most insects, abdominal appendages do not develop prominently; however, some ancestral groups (e.g., Apterygota) retain functional abdominal appendages throughout all developmental stages (i.e., embryonic, nymphal, and adult stages).

In most insects, the abdomen consists of at least 10 segments; however, the boundaries beyond the tenth abdominal segment often become indistinct (Snodgrass 1935). While the segmentation of abdominal segments beyond the tenth remains identifiable in the larvae of holometabolous insects, it is often difficult to discern the segmentation of the terminal abdominal region in both nymphal and adult stages of hemimetabolous insects. In *A. japonicus*, after embryonic development is complete and hatching occurs, the first abdominal segment becomes difficult to observe, and the boundaries between the ninth to eleventh abdominal segments also become indistinct. As a result, it is challenging to infer the precise segmentation of the abdomen based solely on external morphology post‐hatching. However, through detailed observation of embryonic development, it was possible to identify all 11 abdominal segments and trace their developmental processes in detail, as demonstrated in this study. This argument is well consistent with the previous papers (Snodgrass 1935; Tojo and Machida 1997), that discussed the ground plan of abdominal segmentation in insects.

Furthermore, many insect taxa are known to develop abdominal appendages exclusively during embryonic development. During the embryogenesis of *A. japonicus*, paired ventral swellings differentiate in the 1st to 8th abdominal segments at Stage 6. Among these, a pair of pleuropodia forms in a slightly more medial position on the first abdominal segment. Pleuropodia are appendage‐derived structures observed in various insects (e.g., Strauß and Lakes‐Harlan 2006; Viscuso and Sottile 2008). In *A. japonicus*, pleuropodia initially appear as protruding limb primordia at Stage 5 but subsequently invaginate into the embryo during Stage 6, forming glandular structures (Figure 8B’). At this stage, pleuropodia begin secreting enzymes (Figure 11). Similar glandular pleuropodia have been reported in grasshoppers, where they secrete hatching enzymes that help soften the chorion (Slifer 1937, 1938).

In addition, this study observed the formation of spiracles in the 1st to 8th abdominal segments of *A. japonicus*. Spiracles are generally considered to differentiate in a region basal to the coxa of the appendage, particularly in the subcoxa (e.g., Suzuki 1990; Masumoto and Machida 2005; Uchifune and Machida 2005; Komatsu and Kobayashi 2012; Kobayashi et al. 2013). Based on this, the ventral swellings observed in the 1st to 8th abdominal segments (in the first abdominal segment, a swelling structure located slightly lateral to the pleuropodium) correspond to the subcoxal region, while the site where the pleuropodium invaginates in the first abdominal segment corresponds to the coxal region. Additionally, these ventral swellings, like the thoracic appendages, differentiate laterally to the neural ridge. Although the ventral swelling in the first abdominal segment appears slightly more lateral compared to those in the subsequent abdominal segments, this displacement is likely due to the invagination of the pleuropodium, which pushes the swelling outward. However, since its formation occurs between the neural ridge and the tergite, similar to the ventral swellings in the other abdominal segments, it aligns with the differentiation position of the thoracic appendages. These findings further support the hypothesis that the ventral swellings are structures homologous to appendages.

Weber (1952) discussed the evolution of the sternum in arthropods based on morphological, histological, and embryological studies, concluding that the structure of the sternum is derived from the subcoxa of appendages. Similarly, Uchifune and Machida (2005), in their observations of external morphological development during the embryogenesis of *Galloisiana yuasai*, suggested that most of the sternum is derived from the subcoxa of appendages. While this study did not provide definitive evidence regarding whether the sternum in *A. japonicus* is derived from the subcoxa of appendages, it is possible that further detailed investigation into the developmental stages may yield supporting evidence for the subcoxa‐origin hypothesis proposed by Weber (1952) and Uchifune and Machida (2005). Additionally, by observing spiracle formation in the second and third thoracic segments as a criterion, future research may provide a deeper understanding of the homology between thoracic appendages and abdominal swellings.

### 4.3 A Pair of Small Swellings That Differentiates between the Compound Eye Primordium and the Midline of the Head

In the embryogenesis of *A. japonicus*, a pair of small swelling structures forms between the compound eye primordia and the midline of the head at Stage 5 (Figure 14F). To date, no similar structures showing homology to this feature have been reported in other insects or even in other arthropods. This suggests that it is a highly specific structure, likely playing an important role in discussions of segmental organization in the head region of arthropods. In particular, the segmental organization of the preantennal region in arthropods, including insects, remains a subject of considerable uncertainty, even with the use of model organisms in developmental genetic studies. Nevertheless, this structure may offer crucial insights into understanding the segmental organization of the preantennal region.

Although no similar small swellings, as observed in *A. japonicus*, have been reported, in *Tribolium castaneum* (Coleoptera), the *wingless* (*wg*) gene, which defines parasegments, is expressed even anterior to the antennal segment. This has been interpreted as the ocular parasegment (Posnien et al. 2009). Additionally, in a study on *O. fasciatus* (Heteroptera: Lygaeidae) regarding *wg* gene expression, although the authors did not discuss it in detail, one pair of *wg* expression sites anterior to the antennal segment is shown (Angelini and Kaufman 2005). These expression sites are strikingly similar to the position of the small swellings observed in the head region of *A. japonicus*. However, to further investigate this issue, it is necessary not only to observe external morphology but also to examine the configuration of the nervous system in the preantennal segment region through tissue sectioning. Furthermore, elucidating the genetic basis of embryonic development in *A. japonicus* will be crucial for a more comprehensive understanding of this structure.

### 4.4 Mouthparts Development

#### 4.4.1 Labrum (Clypeolabrum)

The labrum is a structure located anterior to the mouthparts in most extant arthropods (Budd 2021). In arthropods with biting‐type mouthparts, the labrum is thought to function as an organ that prevents food from falling forward during feeding (e.g., Robertson and Laverack 1979; De Jong et al. 2002). In contrast, in Hemiptera, the labrum forms a tubular structure that cover the proximal part of the mandibular and maxillary stylets. This structure is exceptionally specialized compared to those of other arthropods and is considered a secondary adaptation to the piercing‐sucking feeding mechanism of hemipteran insects, enabling them to penetrate their food source for nutrient intake.

The clypeolabral primordium in *A. japonicus* begins to differentiate near the anterior end of the presumptive head region at the same developmental stage as the antennal and gnathal appendage primordia (Figure 14B). Initially, it appears as a bilobed structure, resembling the morphology observed during the embryogenesis of *O. fasciatus*, another hemipteran species (Newcomer 1948; Butt 1949). The timing of its differentiation also coincides between these species. Subsequently, in both *A. japonicus* and *O. fasciatus*, the bilobed structure transforms into a single, compact mass and extends posteriorly (Figure 14D–F). In contrast, in *Haplothrips verbasci* (Thysanoptera), a species closely related to Hemiptera that also possesses piercing‐sucking mouthparts, the clypeolabral primordium is reported to form as a single undivided structure from the outset (Heming 1980). The evolutionary origin of the labrum in arthropods has been a subject of long‐standing debate. Some hypotheses propose that it is an appendage‐derived structure of the head (e.g., Snodgrass 1935; Kraus 2001), while others argue that it is a non‐appendicular structure (e.g., Weber 1952; Sharov 1966). Recent studies have attempted to resolve this issue through comparative analyses of gene expression patterns during embryogenesis. However, while some support the appendicular origin hypothesis, specifically that the labrum is derived from the appendages of the intercalary segment (e.g., Haas et al. 2001; Kimm and Prpic 2006), others provide evidence for a non‐appendicular origin (e.g., Posnien et al. 2009). Consequently, no consensus has yet been reached on this matter (Scholtz and Edgecombe 2006).

The observations in this study revealed that the labrum begins to differentiate as a paired structure at the same time as the antennal, gnathal, and thoracic appendage primordia are formed at Stage 4. Based on the timing of its differentiation, it seems to be appendage‐derived; however, the formation near the midline of the embryo suggests that its homologous continuity with appendages in later body segments is weak, indicating that it is a structure distinct from appendages. Although we were unable to observe in detail how the labrum forms a tubular structure in this study, the labrum of first instar nymph of *A. japonicus* is tubular in shape. The mandibular and maxillary stylets pass through this tubular labrum and are housed by the labium, suggesting that the formation of such a structure progresses during Stages 8–9. In *A. japonicus*, from Stage 8 onward, the mandibles and maxillae are covered by the maxillary plates, making further observation difficult. Additionally, since the labrum elongates at the anterior end (Figure 16), it is assumed that the tubular structure reaches its completion after Stage 8.

#### 4.4.2 Mandible, Maxilla and Maxillary Plate

**Figure 18:**
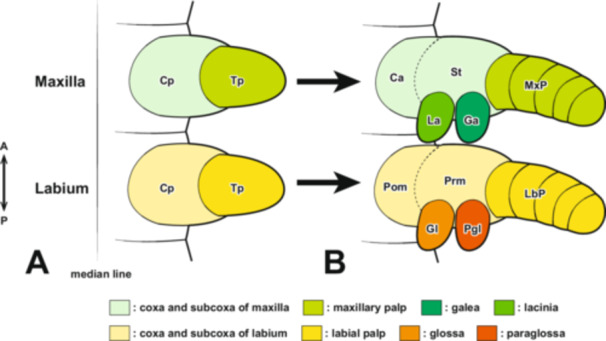
Formation process of the maxilla and labium in insects with generalized biting‐type mouthparts (left half‐body view). In the formation process of the maxilla and labium in generalized insects, each rudiment is initially segmented into a telopodite (Tp) and a coxopodite (Cp) (A). The telopodite subsequently develops into the maxillary palp (MxP) and the labial palp (LbP) (B). The coxopodite of the maxilla further differentiates into the stipes (St) and cardo (Ca). From the stipes, the endite lobes, the inner lobe (lacinia, La) and the outer lobe (galea, Ga), develop. Similarly, in the labium, the coxopodite differentiates into the prementum (Prm) and postmentum (Pom), with the glossa (Gl) and paraglossa (Pgl) developing from the prementum (B). The piercing‐sucking mouthparts of Hemiptera (Figure 1B) are highly specialized, making direct comparisons with generalized biting‐type mouthparts difficult in the fully formed post‐hatching state. However, by examining the developmental process of mouthpart formation during embryogenesis, such comparisons become possible.

In Hemiptera, the mandibles and maxillae are highly specialized, forming stylet‐like structures that differ markedly from those of insects with biting‐type mouthparts. Additionally, hemipteran insects possess a structure known as the “maxillary plate,” which is absent in insects with biting‐type mouthparts. In most insects, the mandibles are considered to lack a telopodite (Snodgrass 1935), meaning that segmentation between the coxopodite and telopodite does not occur. In contrast, the maxillae undergo segmentation into the coxopodite and telopodite, with the telopodite developing into the maxillary palp, while the lacinia and galea differentiate from the inner region of the coxopodite (stipes; Figures 1A and 18; Machida 2000).

There is still no consensus regarding the homology between the maxillary palp, lacinia, and galea in general insects and the corresponding structures in the highly specialized maxillae of Hemiptera. Due to this extreme specialization, differing opinions persist on this issue. Similar to the loss of the telopodite in the mandibles of hemipteran insects, some studies have proposed that the maxillary and labial segments in Hemiptera also lack their respective telopodite‐derived structures, the maxillary palps and labial palps (Snodgrass 1935). Other hypotheses suggest that the maxillary plate originates from the maxillary palps (Dorn and Hoffmann 1983). More recently, a developmental genetic study on *O. fasciatus* suggested that the maxillary stylets are homologous to the maxillary palps, while the maxillary plate is a newly acquired structure distinct from them (Rogers et al. 2002).

In this study, observations of maxillary development in *A. japonicus* revealed that the maxillae undergo segmentation into a coxopodite and a telopodite at Stage 6 (Figure 15A). Subsequently, the telopodite of the maxillae, which corresponds to the maxillary palps in insects with biting‐type mouthparts, enlarges and develops into the maxillary plate. This finding supports the hypothesis proposed by Dorn and Hoffmann (1983) that the maxillary plate originates from the maxillary palps. Rogers et al. (2002), based on the expression patterns of the *distal‐less* (*Dll*) gene in the milkweed bug *O. fasciatus*, the house cricket *Acheta domesticus* (Orthoptera: Gryllidae), and the firebrat *Thermobia domestica* (Zygentoma), suggested that the maxillary stylets are homologous to the maxillary palps, whereas the maxillary plate is a newly acquired structure distinct from them. However, detailed observations of embryonic development in this study clearly demonstrate that the maxillary plate arises from the telopodite (i.e., the maxillary palps), confirming its homology to the maxillary palps. Regarding the *Dll* expression pattern in the maxillae, it is possible that certain insect lineages, which have undergone considerable morphological changes during their evolutionary history, exhibit *Dll* expression patterns that deviate from those traditionally observed in maxillary structures. *Dll* was once regarded as a molecular marker specifically characterizing the telopodite of appendages. However, recent studies have shown that its expression is not exclusive to the telopodite but rather marks structures that extend laterally from the body axis (e.g., Oka et al. 2010; Coulcher and Telford 2012; Bruce and Patel 2020; Matsuoka et al. 2023). The findings of this study provide additional support for these previous observations.

Regarding the maxillary stylets, the morphology of the mouthparts in Psocoptera, the most basal group within the Paraneoptera, suggests a potential evolutionary link between biting‐type and piercing‐sucking‐type mouthparts. Although Psocoptera possess biting‐type mouthparts, their lacinia exhibits a morphology approaching a stylet‐like structure, which has been interpreted as an intermediate trait between these two mouthpart types. This observation suggests that the lacinia may be homologous to the maxillary stylets in Hemiptera (Yoshizawa and Saigusa 2003). Additionally, in Thysanoptera, the maxillary stylets are also considered to be derived from the lacinia (Heming 1980). These findings strongly support the hypothesis that the maxillary stylets in Hemiptera originate from the lacinia. In this study, we were unable to continuously trace the developmental processes of the mandibular and maxillary stylets beyond the stage at which the mandibles and maxillae become obscured within the embryo due to the formation of the maxillary plate. However, in *O. fasciatus*, during the developmental stage at which the elongation of the mandibular and maxillary stylets is externally observable, the maxillary plate is continuous with the basal region of the maxillary stylets (Newcomer 1948). This developmental pattern closely resembles that observed in *H. verbasci* (Thysanoptera) (Heming 1980). Future research on *A. japonicus* should include histological observations, such as serial sectioning, to further elucidate the morphogenesis of the mandibular and maxillary stylets within the embryo.

Similarly, in Thysanoptera, which also possesses piercing‐sucking mouthparts like Hemiptera, a structure resembling the maxillary plate of Hemiptera is observed. However, this structure is thought to be derived from the stipes rather than the maxillary palp. Indeed, a small maxillary palp differentiates from a structure resembling the maxillary plate, which is considered to originate from the stipes (Heming 1980). Additionally, during the embryonic development of *H. verbasci* (Thysanoptera), the maxillary primordium differentiates early, followed by the lateral extension of the stipes, within which the lacinia subsequently differentiates (Heming 1980). These observations suggest that, despite some differences in the mouthpart structures between Thysanoptera and Hemiptera, specialization of the maxillary structures led to the development of an organ that covers the mandibular and maxillary stylets. This may indicate the presence of a common developmental plan underlying the formation of piercing‐sucking mouthparts in these insect groups.

#### 4.4.3 Labium

Traditionally, the labium in *A. japonicus* has been considered to consist of three segments (Tanaka 2001) and has been treated as such in identification keys for belostomatid species (e.g., Hayashi and Miyamoto 2005). However, detailed observations of mouthpart formation in this study revealed that the labium is actually divided into four segments (i.e., one coxopodal segment and three telopodal segments). Nevertheless, in the late stages of embryonic development, the coxopodal region becomes covered by the maxillary plate, giving the appearance of a three‐segmented structure.

Regarding the labium in Hemiptera, although there has not been as much discussion on its homology with the biting‐type mouthparts as there has been for the maxilla, Snodgrass (1935) suggested that the labium in Hemiptera lacks labial palps. However, observations of the labium formation process in this study revealed that, at Stage 6, the labium is segmented into a coxopodite and telopodite, with further segmentation of the telopodite into the first to third telopodal segments of the labium. This finding differs from Snodgrass's suggestion. In typical insect labium formation, after segmentation into the coxopodite and telopodite, the telopodite differentiates into the labial palps, and from the coxopodite, the glossa and paraglossa develop (Figure 18). Therefore, the first to third telopodal segments of the labium in *A. japonicus*, which originate from the telopodite, can be considered homologous to the labial palps, and it is thought that the glossa and paraglossa have degenerated in hemipteran insects. Snodgrass (1935) inferred homology based on completed anatomical structures, but the approach used in this study, which traces the sequential formation process, allows for a more accurate analysis of homology and is considered more reliable. Furthermore, this interpretation aligns with the view of Rogers et al. (2002), who suggested that much of the labium in Hemiptera originates from the labial telopodite, based on the expression patterns of the *proboscipedia* (*pb*) and *Dll* genes, which are important for the formation of labial palps in Diptera and Coleoptera (Beeman et al. 1989, 1993; Hughes and Kaufman 2000; Kaufman 1978; Pultz et al. 1988). On the other hand, regarding the labium in Thysanoptera, which, like in Hemiptera, supports the mandibular and maxillary stylets from beneath, all parts of the labium in Thysanoptera are derived from the labial coxopodite, with a very small labial palp forming at the tip (Heming 1980).

In the labium of *A. japonicus*, an intercalary sclerite differentiates from the tip of the second segment of the labial palps at Stage 8 (Figures 16D and 17B). Although the exact origin of this structure could not be determined in this study, it is an intriguing feature that most likely differentiates from the labial palps.

## 5 Conclusions

As mentioned in the Introduction, one of the key factors behind the species diversity and success of Heteroptera is the acquisition of piercing‐sucking mouthparts, a shared derived trait of this order (Yoshizawa and Saigusa 2003). Systematically, Hemiptera (=Homoptera + Heteroptera) is closely related to Psocoptera and Thysanoptera, which possess intermediate mouthparts between biting and piercing‐sucking. These groups, along with Homoptera and Heteroptera, form the group Paraneoptera (Wang et al. 2013). Among the Paraneoptera, Heteroptera stands out for having highly specialized piercing‐sucking mouthparts, particularly the maxillary plate, a unique structure not found in other insects, and is the most species‐rich group among hemimetabolous insects. This suggests that the acquisition of the maxillary plate may play one of the key roles in the development of highly specialized piercing‐sucking mouthparts in Heteroptera. Specifically, the maxillary plate may have covered the base of the mouthparts (labium), assisting in their movement during feeding and enabling further specialization (e.g., stabilizing the base of the rostrum when piercing a substrate). Because the maxillary plate is an autapomorphy of Heteroptera, its homology with structures in other insects has long been a subject of investigation. Evans (1973) proposed that the maxillary plate is homologous to the stipes in generalized insects. In contrast, Parsons (1964, 1974) described it as a derivative of the genal‐postgenal region. Spangenberg et al. (2013) further emphasized the importance of developmental studies to clarify the evolutionary origin of the maxillary plate. Through detailed observations of embryonic development in *A. japonicus*, this study has shown that the maxillary plate originates from the maxillary palps. These findings suggest that only Heteroptera, which modified the maxillary palps into maxillary plates, were able to acquire highly specialized piercing‐sucking mouthparts. As a result, Heteroptera achieved the highest degree of diversification among other Paraneoptera and hemimetabolous insects. While observations of the embryogenesis of mouthparts in homopteran and other closely related paraneopteran insects are still needed, the results of this study provide important insights into clarifying the previously unclear processes of the acquisition and evolutionary development of piercing‐sucking mouthparts in Heteroptera.

## Author Contributions

**Tomoya Suzuki:** conceptualization, data curation, writing – original draft, scientific illustrations, writing – review and editing. **Takashi Tanizawa:** data curation, providing scientific illustrations, writing – review and editing. **Nobuo Suzuki:** conceptualization, supervision, writing – review and editing. **Koji Tojo:** conceptualization, project administration, resources, supervision, writing – review and editing.

## Conflicts of Interest

The authors declare no conflicts of interest.

### Peer Review

The peer review history for this article is available at https://www.webofscience.com/api/gateway/wos/peer-review/10.1002/jmor.70052.

## Data Availability

The data supporting the findings of this study consist of images created from sectioned samples and SEM samples mounted on stubs. These materials are stored at Shinshu University and Hiroshima Shudo University and are available upon request. All specimens used in this study are reposited in Hiroshima Shudo University.
